# Medical Items

**Published:** 1896-01

**Authors:** 


### *MEDICAL ITEMS.*

EIGHT extra pages in this issue.

---

WE regret to learn of the serious illness of our friend, Dr. Jas. E. Reeves, of Chattanooga. At this writing little hope is entertained as to his recovery.

---

THE latest antiseptic, produced in Germany, is *potassiumorthodinitrocresolate.* It is odorless and cheap, and is said to be destructive to all bacteria. A portion of the potency of this substance is supposed to reside in the name.

---

DR. FRANCIS PEYRE PORCHER, of Charleston, died November 19th. Dr. Porcher was one of the most prominent members of the profession in South Carolina, and for many years had been identified with the Charleston Medical College.

---

A SUBSCRIBER, in renewing his subscription, says: "Your JOURNAL has been a great help to me. I am more and more pleased with it." This good brother appreciates a good thing when he sees it and pays for it cheerfully.

---

DR. J. EDWARD MICHAEL, of Baltimore, died December 7th, in his forty-eighth year. Dr. Michael was for ten years professor of anatomy and clinical surgery in the University of Maryland, and since 1890 professor of obstetrics in the same school. He was highly esteemed as a teacher and a surgeon of ability and distinction.

---

THE medical cuckoo is the American physician that rushes to the public to proclaim to the world the "infallible" value of every sensational fad sprung in a foreign country. When will American physicians (and American girls) overcome their sensational, mawkish yearning for exotic oddities? Let's be original; we have the brains in America.—*North American Medical Review.*

AS SOON as the philologic editor of the *Medical News* and the accomplished editor of the *Medical Record,* and the erudite editor of the *New York Medical Journal,* finish their sparring over the spelling of *uranalysis* and *symphysiotomy* we suggest that they next enlighten their readers upon the status of the latest abomination, *uricacidemia.* These learned gentlemen should examine the credentials of this word and report at once.

---

THE *American Medical Review* is the name o another new journal. It comes from New York, and is edited by Dr. Daniel Lewis. It announces that it begins its career with "definite plans and purposes," without "fads or medical theories." Certain new features are also announced which if properly kept up will make the *Review* popular with medical readers. We congratulate the editor and publishers on the attractive first issue and wish the enterprise an abundance of success.

---

THE committee of the American Medical Association on the department and secretary of public health has appointed an auxiliary steering committee in Washington, consisting of Drs. H. L. Johnson, O. C. Busey, C. H. Kleinschmidt, and J. R. Willington. The Association bill has not been introduced this session, but the old Bureau bill has been introduced in the house. The *Journal of the Association* says that the "Association does not ask for a bureau; it wants a department, and intends to be satisfied with nothing less."

---

BACTERIOLOGISTS are useful assistants, but they are tyrannical masters, and the results of a given treatment must after all be judged, not in the laboratory, but in the hospital ward and the sickroom. A check must be imposed on garrulous bacteriologists who show a disposition to ride the cockhorse among us. We are grateful to them for such assistance as it may be in their power to render to medical science, but we cannot allow them to dictate to us what conclusions we are to draw from clinical investigation. Bacteriological statements are matters of inference, but clinical observations are facts; facts, too, which concern us more nearly than the interesting, but too often contradictory, deductions which foreign laboratory men foist upon us at the point of the scalpel.—*Medical Record.*

---

"WE have been emancipated from the trammels of theory and superstition, and the medicine of to-day is largely based upon exact observation and experimental demonstration. In other words, we may now properly speak of medical science, for, while our knowledge in many directions is far from being complete, it is founded upon a scientific basis of observation and experiment, and is being rapidly extended by scientific methods."—*Sternberg.*

---

ON the third cover page of this issue will be found an announcement which may interest some of our readers, relative to the distribution of six hundred dollars in prizes by the Palisade Manufacturing Company. The prize contest which this well known firm announces will no doubt attract a great deal of attention, and result in the submission of many articles of merit on "The Clinical Value of Antiseptics, Both Internal and External." The prizes are extremely liberal, and the well known professional and literary eminence of Dr. Frank P. Foster, the talented editor of the New York *Medical Journal,* who has kindly consented to act as judge, is a sufficient guarantee of the impartiality to be observed in the awarding of the prizes. We are assured that there is absolutely "no string" attached to the provisions of this contest, and any physician in good standing in the community is invited to compete on equal terms with every other competitor. Further particulars as to conditions, etc., can be obtained by addressing the above named firm.

---

THE Messrs. Appleton & Co., New York, begin this month the publication of *The Journal of Experimental Medicine.* This will be a periodical devoted to original investigations in physiology, pathology, bacteriology, pharmacology, physiological chemistry, hygiene and medicine. This journal is intended to supply the present want of a special medium for the publication of original work in the experimental medical sciences. There are numerous periodicals of this sort in Europe, but none in this country. That the journal will be of high character and representative of scientific medicine in America is assured by the professional standing of those who have promised their co-operation. Among these we notice the names of distinguished professors at Harvard, Yale and Johns Hopkins Universities, the Universities of Pennsylvania and Michigan, Columbia College, etc. The journal will be issued at least in four numbers during the year, probably oftener. The chief editor will be Professor W. H. Welch, Johns Hopkins University, Baltimore.

---

SOME months ago we reviewed in these columns the *Standard Dictionary*, published by the Funk & Wagnalls Company, New York. We note that the company has lately celebrated the first anniversary of the completion of this great work by issuing the 90th thousand. This is quite a number of dictionaries to publish within a year. But the most significant of the triumphs of the first year of this remarkable dictionary, and the most gratifying to Americans, is the wonderful reception given the work by the most exacting of the linguistic critics in England. Especially is this so when we remember how reluctant, naturally enough, the English are to look to a foreign country for a dictionary of their own tongue. It is something extraordinary for an American work of this kind to elicit words of such enthusiastic praise as those uttered by such scholars of the Oxford University as Professor Sayce and Max Muller, and well-known scholars of other English universities, and from such journalistic critics as those of the *London Standard, Saturday Review, Nature, London Times, Westminster Review, Liverpool Post, St. James Budget.* The latter closes his critical review with the following superlative indorsement:

"To say that it is perfect in form and scope is not extravagance of praise, and to say that it is the most valuable dictionary of the English language is but to repeat the obvious. The *Standard Dictionary* should be the pride of literary America, as it is the admiration of literary England."

